# Molecular Detection and Characterization of *Pasteurella multocida* Infecting Camels in Marsabit and Turkana Counties, Kenya

**DOI:** 10.1155/2022/9349303

**Published:** 2022-08-22

**Authors:** Justus K. Kasivalu, George I. Omwenga, Gabriel O. Aboge

**Affiliations:** ^1^Department of Biochemistry, Microbiology and Biotechnology, School of Pure and Applied Sciences, Kenyatta University, Nairobi, Kenya; ^2^Department of Public Health, Pharmacology and Toxicology, Faculty of Veterinary Medicine, University of Nairobi, Nairobi, Kenya

## Abstract

*Pasteurella multocida* infection is common in Kenya though there is little knowledge of the genetic diversity of the pathogen. *P. multocida* is part of the normal flora in the respiratory tract of camels, but it becomes pathogenic when the resistance of the camel body is diminished by bad ecological conditions. This study was conducted to detect, characterize, and determine the genetic diversity of *P. multocida* infecting camels in Marsabit and Turkana Counties. The KMT1 gene was targeted as the marker gene for *P. multocida* and *hyaD-hyaC, bcbD, dcbF, ecbJ*, and *fcbD* as marker genes for capsular serogroups A, B, D, E, and F, respectively. Out of 102 blood and 30 nasal swab samples, twenty-one samples (16%) were confirmed to be positive for *P. multocida* and only capsular group E was detected in both counties. The *P. multocida* sequences were highly conserved and were related to strains from other parts of the world. Our study has confirmed that camels in Marsabit and Turkana Counties of Kenya are infected by *P. multocida* of capsular type E. Farmers should not underfeed camels, ensure appropriate medication and vaccination programs, and minimize herding of camels in crowded areas especially in wet conditions in order to slow the spread of *P. multocida* infection.

## 1. Introduction

Haemorrhagic septicemia (HS) is caused by Pasteurella multocida[1] and is an important disease of camels in Kenya [2]. *P. multocida* can infect different animals and cause different economically important diseases [3]. *P. multocida* causes HS when the camel body is not able to withstand bad ecological conditions such as fluctuations in weather conditions and stress factors such as poor nutrition, walking for a long distance, and parasitic infections [4].

Morbidity associated with *P. multocida* can be low while the mortality rate may be 80% [4]. Camels harboring *P. multocida,* especially young calves, pose the risk of infecting other animals [4]. Transmission of *P. multocida* from infected animal occurs by direct contact and on fomites [1]. Furthermore, transmissions can possibly occur through ingesting or inhaling *P. multocida* emanating from the nasopharynx of infected animals [1]. Where treatment is not given early, the mortality rate can be nearly 100% [1].

HS is broadly distributed, occurring in Southeast Asia, the Middle East, and countries in southern Europe as well as in Japan and North America [1]. HS in Africa happens periodically, does not spread a lot, and is mostly associated with stress conditions [5].

*P. multocida* strains are classified into five capsular serogroups A, B, D, E, and F [6–8]. The biosynthetic loci of the polysaccharide capsule of *P. multocida* have been fully characterized allowing the creation of a PCR-based system that assigns isolates to the five capsule serovars [9]. This typing system involves the hyaD-hyaC gene (involved with hyaluronic acid synthesis) for capsular serogroup A, the dcbF gene (involved in heparin synthesis) for capsular serogroup D, the fcbD gene (involved in chondroitin production) for capsular serogroup F, and the bcbD and ecbJ genes (both involved in glycosyltransferase synthesis) for capsular serogroups B and E, respectively [9].

The diagnosis of HS poses challenges due to the range of clinical appearances and complex laboratory protocols [10]. The detection and characterization of *P. multocida* have depended on the use of traditional biochemical and serological procedures as well as, in recent times, molecular assays [11]. The PCR technique has greatly enhanced *P. multocida* identification [11]. Molecular characterization and understanding of the genetic diversity of *P. multocida* are important for diagnosis, the study of disease epidemiology, the development of vaccines, and the detection of strain variants circulating in a region [12]. This study was carried out to detect *P. multocida* and to characterize the capsular types in diseased camels in Marsabit and Turkana Counties in Kenya.

## 2. Materials and Methods

### 2.1. Study Area and Study Design

**Figure 1:**
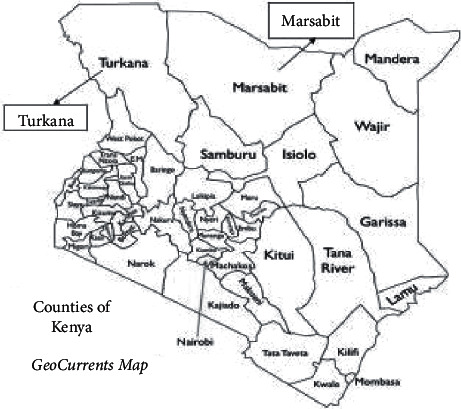
Location of Marsabit and Turkana Counties in Kenya.

Eight sites in two separate counties were investigated, five sites in Marsabit County and three sites in Turkana County. Marsabit County (37°58′E, 2°19′N), situated in northern Kenya, is 70,961 km^2^ in area and has a population of about 459,785. The Marsabit camel population is approximately 132,215. Turkana County (3°09′N 35°21′E), also located in northern Kenya, covers 68,680 km^2^ and has a population of about 926,976. The Turkana camel population is approximately 456,826 [13]. The study area is shown in Figure 1. The study was a retrospective study that used samples collected for diagnostic purposes.

### 2.2. Sample Collection

A total of 132 samples were collected from the two Counties. Sixty-one EDTA blood samples were collected in Marsabit County in November 2018 at five locations, Laisamis-moile, Nairibu, Malabot, El burumagado, and Galas. Another 41 EDTA blood and 30 nasal swab samples were collected in Turkana County in April 2019 at three locations, Nadapal, Lokolia, and Lokore. All samples were from randomly selected camels that had been affected by HS-like disease. The authority to use the blood and nasal swabs from the Central Veterinary Laboratories Kabete was sought from the State Department of Livestock, Directorate of Veterinary Services in Kenya (Permit number MOALF/SDL/DVS/RES/GEN/018). The list of the samples used for the study is shown in Supplementary File 1.

### 2.3. DNA Extraction

Nasal swabs were collected in phosphate-buffered saline (PBS) and transported to the laboratory in a cool box. The nasal swabs were put in tryptic soy broth (Carramore International Limited, Holmfirth, United Kingdom) and incubated at 37°C for 18 hrs, and then stored in a freezer. The frozen whole blood and incubated broths were removed from the freezer and left to thaw at room temperature. The genomic DNA was isolated using PureLink Genomic DNA mini Kit (Invitrogen, Carlsbad, USA) as per the manufacturer's recommendation. DNA was kept at −20°C awaiting PCR analysis.

### 2.4. Detection of *P. multocida* by Conventional PCR and Multiplex PCR

Conventional PCR targeting the KMT1 gene amplified using specific primers [14] was used to detect *P. multocida* (Table 1). *P. multocida* Kabete isolate (A73/17) was used as a positive control and nuclease-free water as a negative control. Amplification was performed by using a Techne TC-512 thermocycler (Bibby Scientific Ltd., Staffordshire, UK). The components for the DNA amplification master mix for *P. multocida* in a total volume of 18.0 *μ*l were nuclease-free water 12.2 *μ*l, 5× PCR buffer 2.5 *μ*l, 10 mM dNTPs 0.5 *μ*l, 10 *μ*M forward primer 0.5 *μ*l, 10 *μ*M reverse primer 0.5 *μ*l, 3 mM MgCl_2_ 1.5 *μ*l, and Taq DNA polymerase 3 units 0.3 *μ*l. The amplification conditions were initial denaturation at 94°C for 3 min followed by 30 cycles of denaturation at 94°C for 45 sec, annealing at 55°C for 45 sec, extension at 72°C for 45 sec, final extension at 72°C for 6 min, and holding at 10°C until removal. A 5 *μ*l aliquot of the PCR product was resolved on a 1.5% agarose gel.

A multiplex PCR targeting capsular genes using specific primer pairs was used on all samples positive in the KMT1 PCR (Table 1) [9]. DNA amplification was carried out in a reaction volume of 20.0 *μ*l that contained nuclease-free water 4.0 *μ*l, 5× PCR buffer 10.0 *μ*l, 10 mM dNTPs 1.25 *μ*l, 10 *μ*M primer pair each 1.25 *μ*l, 3 mM MgCl₂ 1.5 *μ*l, and Taq DNA polymerase 3 units 0.75 *μ*l. Amplification conditions were initial denaturation at 95°C for 5 min followed by 30 cycles of denaturation at 95°C for 1 min, annealing at 55°C for 1 min, extension at 72°C for 1 min, final extension at 72°C for 7 min, holding at 10°C until removal. A 5 *μ*l aliquot of the PCR products was resolved on a 1.5% agarose gel.

### 2.5. DNA Sequencing of *Pasteurella multocida* Amplicons

PCR products of the samples amplified in the assays targeting the KMT1 and *hyaD-hyaC, bcbD, dcbF, ecbJ,* and *fcbD* capsular genes were sequenced at the International Atomic Energy Agency (IAEA) Seibersdorf Laboratories (Vienna, Austria) and Inqaba Biotec (Pretoria, South Africa) using the Sanger sequencing procedure.

### 2.6. Sequence Alignment, Blast Analysis, and Phylogenetic Analysis

Raw sequences were viewed using Chromas version 2.6.6 software [15] and assembled using Geneious prime program version 2020.2.4 software [16] for editing of the assembly and creation of consensus sequence. BioEdit software version 7.2 software [17] was used to import, align, and save aligned sequences. The BLASTn tool of the NCBI GenBank database was used to analyze the sequenced DNA for sequence similarity. The correct species identification was arrived at by comparing the query nucleotides sequences with the available sequences in the GenBank database. The closest BLASTn match was used to confirm the species identification to the homologous sequences found in the GenBank database. *P. multocida* phylogeny was inferred using the maximum likelihood method of MEGA X version 10.1.6 software [18]. Phylogeny was tested using the bootstrap method with a bootstrap of 1,000 repeats and the Tamura–Nei model substitutional method.

## 3. Results

### 3.1. *Pasteurella multocida* Detected by PCR

A positive sample showed the presence of a band of approximately 460 bp. From the Marsabit samples, 15 (25%) had *P. multocida* DNA while in the Turkana samples, 6 (8.5%) yielded *P. multocida* DNA. Overall, 16% (21/132) of the samples from the two counties were positive when amplified with KMT1 primers. Based on the sample type the highest prevalence of *P. multocida* detection was recorded in the blood samples with 18.6% (19/102) and only 6.7% (2/30) for the nasal swabs. The distribution of positives at the various sampling locations is shown in Table 2.

Only the capsular type E gene was detected in all *P. multocida*-positive samples by the multiplex PCR, with all these samples yielding an approximately 511 bp band indicating the presence of the *ecbJ* gene.

### 3.2. *Pasteurella multocida* Confirmed by Sequencing

PCR products from 21 *P. multocida*-positive samples were sequenced. KMT1 gene sequence analysis by the BLASTn tool had 99% to 100% nucleotide similarity to GenBank *P. multocida* nucleotide sequences. BLASTn analysis revealed that the KMT1 gene sequences were identical to the relevant section of the *P. multocida* KMT1 gene. Blast search of the KMT1 gene sequences had a high similarity to accession number LR134532.1. BLASTx search of KMT1 gene sequences for protein matched with a high score of 98% to hydrolase family protein accession number A0085324.1. The sequences for *P. multocida* deposited in GenBank are shown in Supplementary File 2. The PCR products from the capsular typing were sequenced to confirm whether they are of capsular type E, i.e., the *ecbJ* gene. The *ecbJ* gene sequences had a high similarity to accession number AF302466.1 and BLASTx search of *ecbJ* gene sequences for protein matched with a high score of 100% to putative glycosyltransferase EcbJ for capsular group E accession number AAK17912.1. Accession numbers for capsular serogroup E are OL742716 to OL742729.

### 3.3. Phylogenetic Analysis

**Figure 2:**
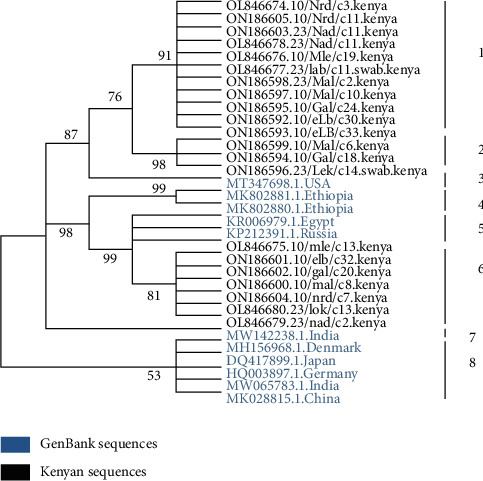
A phylogenetic tree based on the *kmt1* gene was inferred by using the maximum likelihood method and Tamura–Nei model. The tree obtained by neighbor-join and BioNJ algorithms to a matrix of pairwise distances estimated using the Tamura–Nei model. Phylogeny was tested with 1000 bootstrap replications. Evolutionary analyses were conducted in MEGA X version 10.1 software. Numbers indicate clades. Bootstraps are shown at the nodes.

A phylogenetic tree inferred using the maximum likelihood method and based on the KMT1 gene was used to determine whether the 21 *P. multocida* sequences detected in this study were genetically related to those strains from different regions of the world (Figure 2). Eleven Kenyan strains formed clade 1 and another three strains formed clade 2. A strain from the USA formed an outgroup to clades 1 and 2. Ethiopian strains formed clade 4. Strains from Egypt, Russia, and Kenya (all single strains) formed clade 5. Another six Kenyan strains formed clade 6, and one Indian strain formed clade 7 followed by clade 8 formed by strains from Denmark, Japan, Germany, India, and China.

## 4. Discussion

This study was undertaken to detect and then capsule type *P. multocida* in camels raised in the Marsabit and Turkana counties of Kenya. Camel HS is a neglected disease in Kenya, with very few studies focusing on the disease. A number of fungi, bacteria, viruses, and parasites may possibly cause respiratory infection in camels [19]. *P. multocida* causes HS, an acute septicemic disease with high morbidity and mortality in sheep, goat, cattle, and camels, that results in economic losses [1]. Pastoralists in the Lapur division of Turkana district in Kenya identified HS as one of the important camel diseases in Kenya [2]. The community living in Marsabit and Turkana Counties value camels for meat, milk, blood, fat, hides, barter trade, settlement of fines, dowry payment, slaughtering to officiate marriages, performing rituals to appease forefathers, and in ceremonies to elevate elders from one level to another [20]. *P. multocida* was present in the two counties investigated with Marsabit having the highest detection rate. As expected, camels having a HS-like disease were found to be infected with *P.multocida*.

The multiplex PCR applied for capsular typing revealed that all samples positive for *P. multocida* were also positive for *P. multocida* capsular serogroup E. This finding is in agreement with earlier studies, which reported that capsule serogroup B is the characteristic capsular serogroup detected in camels in Iran and while capsular serogroup E is typically reported in Africa [19, 21].

A study conducted in North Kenya by Gluecks et al. [22] focused on HS causing *P.multocida* capsular type B and E from clinically healthy camels that had been affected by HS-like disease. Unlike our study that focused on capsular type A, B, D, E, and F from samples randomly collected from selected camels that had been affected by HS-like disease. Gluecks et al. tested *P. multocida* KMT1-, *psl-,* and KTSP61/KTT72-specific sequences and any sample confirmed positive was subjected to capsular typing for *cap*A, *cap*B, *cap*D, *cap*E, and *cap*F while our study tested KMT1-specific sequences and subjected the positives to capsular typing for the same capsular types according to Townsend et al. [9]. In their study, they confirmed that haemorrhagic septicemia-specific sequences KTSP61/KTT72 lacked HS-associated capsular type B and E genes *cap*B and *cap*E that were not used in our study, but we were able to detect capsular type E gene *cap*E by Townsend et al. protocol [9].

Gluecks et al. concluded there was a circulation of HS strain in camels that lack established capsular types contrary to our study where we report the presence of capsular type E in circulation in Marsabit and Turkana Counties. Furthermore, their study confirmed the presence of *P. multocida* capsular type A- and D-specific DNA sequences though at very low frequency making their molecular study to be the first to confirm the presence of the two capsular types. The findings of our study confirm that capsular serogroup E is mainly responsible for haemorrhagic septicemia in camels in Kenya.

The alignment of the Kenyan *P. multocida* KMT1 gene sequences with 11 sequences retrieved from GenBank using BioEdit software version 7.2 [17] revealed that the Kenyan strains were highly conserved. The KMT1 gene of the Kenyan strains clustered into different clades from those associated with strains from other regions of the world, indicating the relative uniqueness of the *P. multocida* strains circulating in Marsabit and Turkana Counties in Kenya.

Only capsular group E was detected, other capsular groups A, B, D, and F were not detected. There is a certain relationship between *P. multocida* capsular group, diseases, and host species, which is supported by the geographical distribution of capsular groups [23]. There has been some deviation from these relationships in recent years [24].

In this study, 21/132 samples were found to be positive for *P. multocida* and in capsular typing, they were found to be capsular type E. Multiplex PCR provided a quick replacement to available phenotypic tests for the identification of capsulated *P. multocida* because it allows the concomitant, quick detection of genes, and capsular typing [25, 26].

## 5. Conclusion

Our study has confirmed that the camels in Marsabit and Turkana Counties of Kenya were infected by *P. multocida* of capsular type E. Farmers should provide camels with appropriate nutrition, properly treat and vaccinate camels, minimize herding of camels in crowded areas especially in wet conditions to slow the fast spread of *P. multocida* infection. We recommend further research on HS, *P. multocida* genetic diversity, and the molecular epidemiology of this disease agent.

## Figures and Tables

**Table 1:** Sequences of oligonucleotide primers used for identification and capsular typing of *P. multocida.*

Gene	Primer (5′–3′)	Amplicon size (bp)	Reference
*Kmt1*	ATCCGCTATTTACCCAGTGG	460	[14]
	GCTGTAAACGAACTCGCCAC		

*hyaD-hyaC*	TGCCAAAATCGCAGTGAG	1044	[9]
	TTGCCATCATTGTCAGTG		

*bcbD*	CATTTATCCAAGCTCCACC	760	[9]
	GCCCGAGAGTTTCAATCC		

*dcbF*	TTACAAAAGAAAGACTAGGAGCCC	657	[9]
	CATCTACCCACTCAACCATATCAG		

*ecbJ*	TCCGCAGAAAATTATTGACTC	511	[9]
	GCTTGCTGCTTGATTTTGTC		

*fcbD*	AATCGGAGAACGCAGAAATCAG	851	[9]
	TTCCGCCGTCAATTACTCTG		

**Table 2:** Results of PCR analysis of the samples collected for the study^*∗*^.

County	Location	Type of sample	No. of samples	No. of samples positive
Marsabit	Laisamis-moile	Blood	10	2
	Nairibu	Blood	12	3
	Malabot	Blood	16	4
	El Burumagado	Blood	8	3
	Galas	Blood	15	3

Turkana	Nadapal	Blood	6	3
		Nasal swab	5	0
	Lokolia	Blood	15	1
		Nasal swab	15	1
	Lokore	Blood	20	0
		Nasal swab	10	1
		Total	132	21

^*∗*^All *P. multocida* positives were found to be capsule type E.

## Data Availability

The DNA sequence data used to support the findings of this study have been deposited in the NCBI under accession numbers OL742716–OL742729, OL846674–OL846680, and ON186592–ON186605.

## Supplementary Materials

Supplementary Materials Supplementary File 1: Marsabit and Turkana samples used for the study. Supplementary File 2: accession numbers of DNA sequences for *P.multocida* deposited in GenBank.
