# Supplementary material for: Molecular Detection and Characterization of Pasteurella multocida Infecting Camels in Marsabit and Turkana Counties, Kenya
Source: Int J Microbiol. 2022 Aug 22;2022:9349303. doi: 10.1155/2022/9349303 (PMC9424043; doi:10.1155/2022/9349303)
Supplement: Supplementary Materials — Supplementary File 1: Marsabit and Turkana samples used for the study. Supplementary File 2: accession numbers of DNA sequences for P.multocida deposited in GenBank. [file 9349303.f1.zip › 9349303.f1/Supplementary File 1. Marsabit and Turkana samples used for the study.docx]

**Supplementary File 1**. Marsabit and Turkana samples used for the study

| No. | Camel ID | County | Location | Type of sample | Age (Yr) | Sex | clinical status |
| --- | --- | --- | --- | --- | --- | --- | --- |
| 1 | 10/NRB/C1 | Marsabit | Nairibu | Blood | Adult | Castrate | Nasal discharge, fever, labored respiration |
| 2 | 10/NRB/C2 | Marsabit | Nairibu | Blood | Adult | F | Nasal discharge, fever, labored respiration |
| 3 | 10/NRB/C3 | Marsabit | Nairibu | Blood | Adult | F | Nasal discharge, fever, labored respiration |
| 4 | 10/NRB/C4 | Marsabit | Nairibu | Blood | Adult | F | Nasal discharge, fever, labored respiration |
| 5 | 10/NRB/C5 | Marsabit | Nairibu | Blood | Adult | F | heathy |
| 6 | 10/NRB/C6 | Marsabit | Nairibu | Blood | Adult | M | heathy |
| 7 | 10/NRB/C7 | Marsabit | Nairibu | Blood | Adult | F | heathy |
| 8 | 10/NRB/C8 | Marsabit | Nairibu | Blood | Adult | F | heathy |
| 9 | 10/NRB/C9 | Marsabit | Nairibu | Blood | Adult | M | fever, nasal discharge |
| 10 | 10/NRB/C10 | Marsabit | Nairibu | Blood | Adult | F | fever, nasal discharge |
| 11 | 10/NRB/C11 | Marsabit | Nairibu | Blood | Adult | F | healthy |
| 12 | 10/NRB/C12 | Marsabit | Nairibu | Blood | Adult | F | mange infested |
| 13 | 10/MLE/C13 | Marsabit | Moile | Blood | 4 | F | treated for trypanosomes with abscess |
| 14 | 10/MLE/C14 | Marsabit | Moile | Blood | 1.3 | F | treated for trypanosomes with abscess |
| 15 | 10/MLE/C15 | Marsabit | Moile | Blood | 4 | F | treated for trypanosomes with abscess |
| 16 | 10/MLE/C16 | Marsabit | Moile | Blood | 3 | M | treated for trypanosomes with abscess |
| 17 | 10/MLE/C17 | Marsabit | Moile | Blood | 21 | F | treated for trypanosomes with abscess |
| 18 | 10/MLE/C18 | Marsabit | Moile | Blood | 2 | M | treated for trypanosomes with abscess |
| 19 | 10/MLE/C19 | Marsabit | Moile | Blood | 1 | M | treated for trypanosomes with abscess |
| 20 | 10/MLE/C20 | Marsabit | Moile | Blood | 2 | M | with very swollen prescapular lymph node |
| 21 | 10/MLE/C21 | Marsabit | Moile | Blood | 2 | F | with very swollen prescapular lymph node |
| 22 | 10/ELB/C26 | Marsabit | El-borumagado | Blood | 17 | F | treated for trypanosomes, fever |
| 23 | 10/ELB/C27 | Marsabit | El-borumagado | Blood | Adult | F | treated for trypanosomes, fever |
| 24 | 10/ELB/C28 | Marsabit | El-borumagado | Blood | Adult | F | treated for trypanosomes, fever |
| 25 | 10/ELB/C29 | Marsabit | El-borumagado | Blood | Adult | F | treated for trypanosomes, fever |
| 26 | 10/ELB/C30 | Marsabit | El-borumagado | Blood | 1 | F | treated for trypanosomes, fever |
| 27 | 10/ELB/C31 | Marsabit | El-borumagado | Blood | Adult | F | treated for trypanosomes, fever |
| 28 | 10/ELB/C32 | Marsabit | El-borumagado | Blood | Adult | F | treated for trypanosomes, fever |
| 29 | 10/ELB/C33 | Marsabit | El-borumagado | Blood | Adult | M | treated for trypanosomes, fever |
| 30 | 10/MAL/C1 | Marsabit | Malabot | Blood | 22 | F | swollen prescapular lymph node, fever |
| 31 | 10/MAL/C2 | Marsabit | Malabot | Blood | 9 | Castrate | fever, nasal discharge, labored respiration |
| 32 | 10/MAL/C3 | Marsabit | Malabot | Blood | 16 | F | labored respiration, fever |
| 33 | 10/MAL/C4 | Marsabit | Malabot | Blood | 20 | F | ocular discharge, labored respiration |
| 34 | 10/MAL/C5 | Marsabit | Malabot | Blood | 2 | F | Fever, ocular discharge |
| 35 | 10/MAL/C6 | Marsabit | Malabot | Blood | 9 | F | labored respiration, hypersalivation, fever |
| 36 | 10/MAL/C7 | Marsabit | Malabot | Blood | 2 | F | ocular discharge, hypersalivation |
| 37 | 10/MAL/C8 | Marsabit | Malabot | Blood | 1 | F | treated for trypanosomes with abscess |
| 38 | 10/MAL/C9 | Marsabit | Malabot | Blood | 1 | M | treated for trypanosomes with abscess |
| 39 | 10/MAL/C10 | Marsabit | Malabot | Blood | 1 | F | treated for trypanosomes with abscess |
| 40 | 10/MAL/C11 | Marsabit | Malabot | Blood | 5 | F | labored respiration, hypersalivation, fever |
| 41 | 10/MAL/C12 | Marsabit | Malabot | Blood | 10 | F | labored respiration, hypersalivation, fever |
| 42 | 10/MAL/C13 | Marsabit | Malabot | Blood | 3 | F | Fever, ocular discharge |
| 43 | 10/MAL/C14 | Marsabit | Malabot | Blood | 2 | F | Fever, ocular discharge, diarrhea |
| 44 | 10/MAL/C15 | Marsabit | Malabot | Blood | 3 | F | Fever, ocular discharge, diarrhea |
| 45 | 10/MAL/C16 | Marsabit | Malabot | Blood | 16 | F | Fever, ocular discharge, diarrhea |
| 46 | 10/MAL/C17 | Marsabit | Malabot | Blood | 3 month | M | treated for trypanosomes with abscess |
| 47 | 10/GAL/C18 | Marsabit | Galas | Blood | 5 | F | treated for trypanosomes with abscess, fever |
| 48 | 10/GAL/C19 | Marsabit | Galas | Blood | 5 | F | healthy |
| 49 | 10/GAL/C20 | Marsabit | Galas | Blood | 7 | F | healthy |
| 50 | 10/GAL/C21 | Marsabit | Galas | Blood | 5 | F | Nasal discharge |
| 51 | 10/GAL/C22 | Marsabit | Galas | Blood | 12 | F | Swollen lymph node |
| 52 | 10/GAL/C23 | Marsabit | Galas | Blood | 13 | F | healthy |
| 53 | 10/GAL/C24 | Marsabit | Galas | Blood | 6 | F | salivation |
| 54 | 10/GAL/C25 | Marsabit | Galas | Blood | 10 | F | fever |
| 55 | 10/GAL/C34 | Marsabit | Galas | Blood | 6 | M | diarrhea, ocular discharge |
| 56 | 10/GAL/C35 | Marsabit | Galas | Blood | 12 | F | Nasal discharge |
| 57 | 10/GAL/C36 | Marsabit | Galas | Blood | 4 | F | healthy |
| 58 | 10/GAL/C37 | Marsabit | Galas | Blood | 7 | F | diarrhea |
| 59 | 10/GAL/C38 | Marsabit | Galas | Blood | 7 | F | Fever, ocular discharge, diarrhea |
| 60 | 10/GAL/C39 | Marsabit | Galas | Blood | 5 | M | healthy |
| 61 | 10/GAL/C40 | Marsabit | Galas | Blood | 22 | F | Ocular discharge |
| 62 | 23/NAD/C1 | Turkana | Nadapal | Blood | Weaner | F | Ocular discharge |
| 63 | 23/NAD/C2 | Turkana | Nadapal | Blood | Weaner | M | Ocular discharge |
| 64 | 23/NAD/C3 | Turkana | Nadapal | Blood | Weaner | M | Ocular discharge |
| 65 | 23/NAD/C4 | Turkana | Nadapal | Blood | Adult | F | Ocular discharge |
| 66 | 23/NAD/C5 | Turkana | Nadapal | Blood | Adult | F | Ocular discharge, |
| 67 | 23/NAD/C6 | Turkana | Nadapal | Blood | Adult | F | Ocular discharge, fever |
| 68 | 23/NAD/C7 | Turkana | Nadapal | Blood | Adult | F | Ocular discharge |
| 69 | 23/NAD/C8 | Turkana | Nadapal | Blood | Adult | F | Ocular discharge |
| 70 | 23/NAD/C9 | Turkana | Nadapal | Blood | Adult | F | Ocular discharge |
| 71 | 23/NAD/C10 | Turkana | Nadapal | Blood | Adult | F | Ocular discharge |
| 72 | 23/NAD/C11 | Turkana | Nadapal | Blood | Adult | F | Ocular discharge |
| 73 | 23/LOK/C1 | Turkana | Lokolia | Blood | Adult | F | healthy |
| 74 | 23/LOK/C2 | Turkana | Lokolia | Blood | Adult | F | Abscess, Ocular discharge |
| 75 | 23/LOK/C3 | Turkana | Lokolia | Blood | Adult | F | Fever, ocular discharge |
| 76 | 23/LOK/C4 | Turkana | Lokolia | Blood | Adult | F | healthy |
| 77 | 23/LOK/C5 | Turkana | Lokolia | Blood | Weaner | F | healthy |
| 78 | 23/LOK/C6 | Turkana | Lokolia | Blood | Adult | F | diarrhea, hypersalivation |
| 79 | 23/LOK/C7 | Turkana | Lokolia | Blood | Adult | F | ocular discharge, recumbency |
| 80 | 23/LOK/C8 | Turkana | Lokolia | Blood | Adult | F | recumbency,diarrhea, hypersalivation |
| 81 | 23/LOK/C9 | Turkana | Lokolia | Blood | Adult | F | healthy |
| 82 | 23/LOK/C10 | Turkana | Lokolia | Blood | Adult | F | ocular discharge, fever |
| 83 | 23/LOK/C11 | Turkana | Lokolia | Blood | Adult | M | diarrhea, hypersalivation |
| 84 | 23/LOK/C12 | Turkana | Lokolia | Blood | Calf | F | healthy |
| 85 | 23/LOK/C13 | Turkana | Lokolia | Blood | Adult | F | healthy |
| 85 | 23/LOK/C14 | Turkana | Lokolia | Blood | Adult | F | healthy |
| 87 | 23/LOK/C15 | Turkana | Lokolia | Blood | Weaner | M | diarrhea, hypersalivation |
| 88 | 23/LOR/C1 | Turkana | Lokore | Blood | Weaner | M | treated for trypanosomes with abscess |
| 89 | 23/LOR/C2 | Turkana | Lokore | Blood | Adult | F | fever |
| 90 | 23/LOR/C3 | Turkana | Lokore | Blood | Adult | F | Nasal discharge` |
| 91 | 23/LOR/C4 | Turkana | Lokore | Blood | Adult | F | swollen lymph node |
| 92 | 23/LOR/C5 | Turkana | Lokore | Blood | Adult | F | salivation |
| 93 | 23/LOR/C6 | Turkana | Lokore | Blood | Adult | F | fever, ocular discharge |
| 94 | 23/LOR/C7 | Turkana | Lokore | Blood | Adult | F | healthy |
| 95 | 23/LOR/C8 | Turkana | Lokore | Blood | Adult | F | diarrhea |
| 96 | 23/LOR/C9 | Turkana | Lokore | Blood | Adult | F | fever, salivation |
| 97 | 23/LOR/C10 | Turkana | Lokore | Blood | Adult | F | labored respiration |
| 98 | 23/LOR/C11 | Turkana | Lokore | Blood | Weaner | F | fever, salivation |
| 99 | 23/LOR/C12 | Turkana | Lokore | Blood | Adult | F | healthy |
| 100 | 23/LOR/C13 | Turkana | Lokore | Blood | weaner | F | healthy |
| 101 | 23/LOR/C14 | Turkana | Lokore | Blood | Adult | F | healthy |
| 102 | 23/LOR/C15 | Turkana | Lokore | Blood | Adult | F | healthy |
| 103 | 23/NAD/C1 | Turkana | Nadapal | Swab | weaner | F | Nasal discharge, fever |
| 104 | 23/NAD/C2 | Turkana | Nadapal | Swab | weaner | M | Nasal discharge, salivation |
| 105 | 23/NAD/C4 | Turkana | Nadapal | Swab | weaner | M | Nasal discharge, labored respiration |
| 106 | 23/NAD/C8 | Turkana | Nadapal | Swab | Adult | F | Nasal discharge, fever |
| 107 | 23/NAD/C11 | Turkana | Nadapal | Swab | Adult | F | Nasal discharge, ocular discharge |
| 108 | 23/LOK/C1 | Turkana | Lokolia | Swab | Adult | F | Nasal discharge, ocular discharge |
| 109 | 23/LOK/C2 | Turkana | Lokolia | Swab | Adult | F | Nasal discharge, fever |
| 110 | 23/LOK/C3 | Turkana | Lokolia | Swab | Adult | F | Nasal discharge, fever, ocular discharge |
| 111 | 23/LOK/C4 | Turkana | Lokolia | Swab | Adult | F | Nasal discharge, labored respiration |
| 112 | 23/LOK/C5 | Turkana | Lokolia | Swab | Weaner | F | Nasal discharge, fever, salivation |
| 113 | 23/LOK/C6 | Turkana | Lokolia | Swab | Adult | F | Nasal discharge, ocular discharge |
| 114 | 23/LOK/C7 | Turkana | Lokolia | Swab | Adult | F | Nasal discharge, salivation |
| 115 | 23/LOK/C8 | Turkana | Lokolia | Swab | Adult | F | Nasal discharge, fever |
| 116 | 23/LOK/C9 | Turkana | Lokolia | Swab | Adult | F | Nasal discharge, treated for trypanosomes |
| 117 | 23/LOK/C10 | Turkana | Lokolia | Swab | Adult | F | Nasal discharge, fever, treated for trypanosomes |
| 118 | 23/LOK/C11 | Turkana | Lokolia | Swab | Adult | F | Nasal discharge, ocular discharge, fever |
| 119 | 23/LOK/C12 | Turkana | Lokolia | Swab | calf | F | Nasal discharge, labored respiration, fever |
| 120 | 23/LOK/C13 | Turkana | Lokolia | Swab | Adult | F | Nasal discharge, fever, salivation |
| 121 | 23/LOK/C14 | Turkana | Lokolia | Swab | Weaner | F | Nasal discharge, Abscess, fever |
| 122 | 23/LOK/C15 | Turkana | Lokolia | Swab | Weaner | M | Nasal discharge, Abscess, fever |
| 123 | 23/LOR/C2 | Turkana | Lokore | Swab | Adult | F | Nasal discharge, swollen lymph node |
| 124 | 23/LOR/C3 | Turkana | Lokore | Swab | Adult | F | Nasal discharge, fever, swollen lymph node |
| 125 | 23/LOR/C4 | Turkana | Lokore | Swab | Adult | F | Nasal discharge, ocular discharge |
| 126 | 23/LOR/C5 | Turkana | Lokore | Swab | Adult | F | Nasal discharge, diarrhea, fever |
| 127 | 23/LOR/C6 | Turkana | Lokore | Swab | Adult | F | Nasal discharge, diarrhea, fever |
| 128 | 23/LOR/C7 | Turkana | Lokore | Swab | Adult | F | Nasal discharge, diarrhea, fever |
| 129 | 23/LOR/C8 | Turkana | Lokore | Swab | Adult | F | Nasal discharge, labored respiration |
| 130 | 23/LOR/C9 | Turkana | Lokore | Swab | Adult | F | Nasal discharge |
| 131 | 23/LOR/C10 | Turkana | Lokore | Swab | Adult | F | Nasal discharge |
| 132 | 23/LOR/C11 | Turkana | Lokore | Swab | Weaner | F | Nasal discharge |
