# Supplementary material for: Molecular Detection and Characterization of Pasteurella multocida Infecting Camels in Marsabit and Turkana Counties, Kenya
Source: Int J Microbiol. 2022 Aug 22;2022:9349303. doi: 10.1155/2022/9349303 (PMC9424043; doi:10.1155/2022/9349303)
Supplement: Supplementary Materials — Supplementary File 1: Marsabit and Turkana samples used for the study. Supplementary File 2: accession numbers of DNA sequences for P.multocida deposited in GenBank. [file 9349303.f1.zip › 9349303.f1/Supplementary File 2. Accession numbers of DNA sequences for P.multocida deposited in GenBank..docx]

| Sample ID | Target Gene | Accession number |
| --- | --- | --- |
| 10/NRB/C3 | kmt1 | OL846674 |
| 10/NRB/C7 | kmt1 | ON186604 |
| 10/NRB/C11 | kmt1 | ON186605 |
| 10/MLE/C13 | kmt1 | OL846675 |
| 10/MLE/C19 | kmt1 | OL846676 |
| 10/ELB/C30 | kmt1 | ON186592 |
| 10/ELB/C32 | kmt1 | ON186601 |
| 10/ELB/C33 | kmt1 | ON186593 |
| 10/MAL/C2 | kmt1 | ON186598 |
| 10/MAL/C6 | kmt1 | ON186599 |
| 10/MAL/C8 | kmt1 | ON186600 |
| 10/MAL/C10 | kmt1 | ON186597 |
| 10/GAL/C18 | kmt1 | ON186594 |
| 10/GAL/C20 | kmt1 | ON186602 |
| 10/GAL/C24 | kmt1 | ON186595 |
| 23/NAD/C1 | kmt1 | OL846678 |
| 23/NAD/C2 | kmt1 | OL846679 |
| 23/NAD/C11 | kmt1 | ON186603 |
| 23/LOK/C13 | kmt1 | OL846680 |
| 23/LOK/C14 | kmt1 | ON186596 |
| 23/LOR/C11 | kmt1 | OL846677 |

Supplementary File 2. Accession numbers of DNA sequences for *P.multocida* deposited in GenBank.
